# Ketamine decreases sensitivity of male rats to misleading negative feedback in a probabilistic reversal-learning task

**DOI:** 10.1007/s00213-016-4497-1

**Published:** 2016-12-08

**Authors:** Michal Rychlik, Eva Bollen, Rafal Rygula

**Affiliations:** Institute of Pharmacology, Polish Academy of Sciences, Department of Behavioral Neuroscience and Drug Development, Affective Cognitive Neuroscience Laboratory, 12 Smetna Street, 31-343 Krakow, Poland

**Keywords:** Rat, Feedback sensitivity, Probabilistic reversal learning, Ketamine, Animal model

## Abstract

**Rationale:**

Depression is characterized by an excessive attribution of value to negative feedback. This imbalance in feedback sensitivity can be measured using the probabilistic reversal-learning (PRL) task. This task was initially designed for clinical research, but introduction of its rodent version provides a new and much needed translational paradigm to evaluate potential novel antidepressants.

**Objectives:**

In the present study, we aimed at evaluating the effects of a compound showing clear antidepressant properties—ketamine (KET)—on the sensitivity of rats to positive and negative feedback in the PRL paradigm.

**Methods:**

We trained healthy rats in an operant version of the PRL task. For successful completion of the task, subjects had to learn to ignore infrequent and misleading feedback, arising from the probabilistic (80:20) nature of the discrimination. Subsequently, we evaluated the effect of KET (5, 10, and 20 mg/kg) on feedback sensitivity 1, 24, and 48 h after administration.

**Results:**

We report that acute administration of the highest dose of KET (20 mg/kg) rapidly and persistently decreases the proportion of lose–shift responses made by rats after receiving negative feedback.

**Conclusion:**

Present results suggest that KET decreases negative feedback sensitivity and that changes in this basic neurocognitive function might be one of the factors responsible for its antidepressant action.

## Introduction

In everyday life, we constantly experience events that we associate with a specific emotional value. These acquired cognitive-affective associations help us predict the emotional outcome of future events and as such, direct our behavior. Depressive patients show an aberrant pattern of cognitive–affective directed behaviors, i.e., positive events often remain undervalued when making future responses, whereas there is an excessive sensitivity to negative events (Eshel and Roiser 2010). This leads to a state in which pleasurable stimuli are no longer rewarding, and in which there is a particular focus on past negative events, as well as on potential negative outcomes of future behavior.

This negative bias in feedback sensitivity can be measured using a probabilistic reversal-learning (PRL) paradigm (Cools et al. 2002). It is different from normal reversal learning in that subjects should ignore misleading negative and positive feedback to maximize reward and minimize punishment. Using this task, it has been demonstrated that depressed patients show increased value attribution towards negative events (Taylor Tavares et al. 2008), and that other disorders are also associated with impaired PRL performance, including schizophrenia and Parkinson’s disease (Peterson et al. 2009; Waltz and Gold 2007). In 2010, an innovative PRL procedure for testing feedback sensitivity in rodents was described for the first time (Bari et al. 2010). With current classical depression-related behavioral paradigms (e.g., tail-suspension test and forced swim test) lacking face and ecological validity, and the growing need for bridging the gap between preclinical and clinical studies being recognized in projects such as National Institute of Mental Health Research Domain Criteria (RDoC) initiative (Morris and Cuthbert 2012), this novel operant task is a very compelling tool for preclinical tests of the impact of drugs on cognitive–affective systems; dysfunction of which is observed in depression (Dzirasa and Covington 2012; Pryce and Seifritz 2011).

In the recent years, the area of antidepressant research has shifted from the stereotypical monoaminergic agents to novel targets, including the glutamatergic system (O’Leary et al. 2015; Serafini et al. 2013; Skolnick et al. 1996; Skolnick et al. 2009). One particularly promising strategy to alleviate depressive-like symptoms is by targeting glutamatergic *N*-methyl-D-aspartate receptor (NMDAR) signaling. The uncompetitive NMDAR antagonist ketamine (KET), which is mostly known as an anesthetic, has shown rapid antidepressant effects in humans (aan het Rot et al. 2010; Zarate et al. 2006) and animals (Garcia et al. 2008; Yilmaz et al. 2002).

KET provides a major advantage over the widely prescribed monoaminergic antidepressants, such as selective serotonin reuptake inhibitors (SSRIs), because it shows acute antidepressant effects, as assessed by self-report questionnaires (for example: Beck Depression Inventory or Montgomery–Åsberg Depression Rating Scale), whereas SSRIs require chronic treatment of 2 to 3 weeks before reaching full effect. However, investigation of the effects of monoaminergic antidepressants on affective processing of information showed that even when administered acutely, these compounds modulate cognitive–affective functions in depressed patients (Harmer et al. 2009), healthy volunteers (Harmer et al. 2003), and experimental animals (Bari et al. 2010; Ineichen et al. 2012). This observation has led to a hypothesis stating that the delay in antidepressant action of monoaminegric drugs reflects the time necessary to adjust ones cognitive schemes to newly experienced reality (Roiser et al. 2012). Consequently, according to this theory, KET should not only balance affective processing of information but also facilitate relearning, i.e., cognitive functions, and human studies provide some evidence for the latter (Murrough et al. 2015). Nonetheless, to our knowledge, there have been no studies conducted to assess KET’s impact on affective neuropsychological functions.

Therefore, in this study, we have evaluated the effects of KET on feedback sensitivity. Due to its antidepressant profile, we hypothesized that KET would decrease negative feedback sensitivity (NFS).

## Materials and methods

### Subjects and housing

We used 32 male Sprague Dawley rats (Charles River, Germany), weighing between 175 and 200 g upon arrival. We housed the rats in groups (4 animals/cage) in a temperature (21 ± 1 °C) and humidity (40–50%) controlled room. The rats were kept under a 12/12-h light/dark (L/D) cycle (lights on at 07:00 h). The animals were mildly food restricted to approximately 85% of their free-feeding weights, which was achieved by providing 15–20 g of food per rat per day (standard laboratory chow, Labofeed, Kcynia, Poland). Food was restricted beginning at 1 week prior to training. Water was freely available, with the exception of during the test sessions. All the behavioral procedures were performed during the light phase of the L/D cycle.

### Apparatus

The behavioral tests were performed in eight computer-controlled operant conditioning boxes (Med Associates, St Albans, Vermont, USA); each box was equipped with a light, a speaker, a liquid dispenser (set to deliver 0.1 ml of 20% sucrose solution), and two retractable levers. The levers were located on opposite sides of the feeder. All of the behavioral protocols, including the data acquisition and recordings, were programmed in Med State notation code (Med Associates).

### Initial instrumental training

The animals were trained to perform the probabilistic reversal-learning task in four stages. In the first stage, the animals were trained to recognize and collect the reward. To this end, every 10 s, rats were presented with the reward (0.1 ml 20% sucrose solution) for 5 s. These training sessions lasted 30 min.

In the second stage, one of the levers (left/right counterbalanced between stages/animals) was constantly extended and every press on this lever was rewarded with 5-s presentation of the reward (fixed ratio schedule of reinforcement 1:1). The animals were trained to a criterion of 60 presses in 30 min.

After learning the lever-reward association, during stage 3, the rats were familiarized with intertrial intervals (ITIs). In this stage, the lever (left/right counterbalanced between stages/animals) was retracted after collection of the reward and house light was switched off for 3 s (ITI) before the next trial commenced. No response within 10 s from lever presentation was marked as an omission and a criterion of less than 20% omissions had to be met before progressing to the fourth stage of the training.

The last, fourth stage of the training consisted of random presentations of either left or right levers; each of which had to be pressed at least 30 times in 30 min. To avoid side bias during the PRL task, animals had to respond with similar frequency on both levers. This was achieved by training the rats to a criterion of less than 7.5% omissions on each lever (i.e., less than 15% total omissions but equally distributed between the levers) for 3 consecutive training days. After attaining this criterion the animals were ready to be tested in the PRL procedure.

### Training for the PRL task

**Fig. 1:**
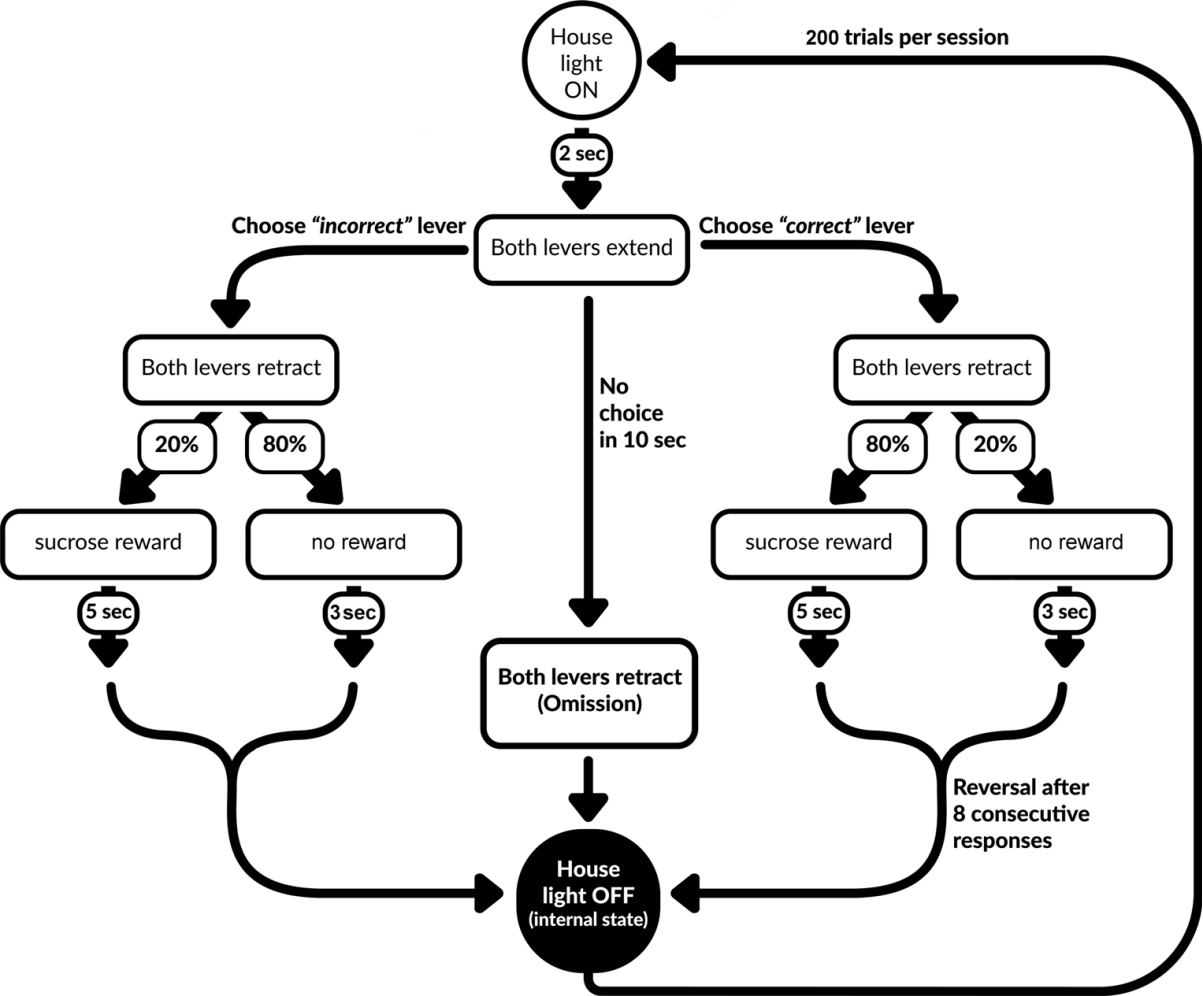
Schematic overview of the PRL task

The PRL procedure used in our study was a modified version of the procedures described by Bari et al. (2010) and Dalton et al. (2014) (see Fig. 1 for a schematic overview of the task). A PRL training session consisted of 200 trials. Each trial lasted for a maximum of 20 s. The start of a trial was signaled by the house light, which remained on until the end of a trial. Two seconds after the trial had started, both levers were presented and one of them was randomly assigned as the “correct” lever, which delivered reward on 80% of the times it was pressed. A press on the other—“incorrect”—lever would result in a rewarding outcome with a probability of only 20%. No response in 10 s triggered the ITI and was counted as an omission.

The same ITI directly followed a punishing outcome, i.e., no reward on 20% of the correct and 80% of the incorrect lever presses. After every 8 consecutive correct lever presses (regardless of the outcome), a criterion for a reversal of the outcome probabilities was reached. Previously correct lever now became incorrect and vice versa. This pattern was followed until the end of a session.

The number of reversals completed was the main measure of interest for the evaluation of the PRL training process.

This training phase was repeated daily until individual animals achieved sufficient performance levels. The criteria to be met were a minimum of 2 reversals completed during 3 consecutive training sessions, with a difference of no more than 1 reversal completed between 2 consecutive days. Baseline performance for all behavioral measures was calculated as the mean from these 3 criterion sessions.

### PRL testing and behavioral measures of interest

The procedures in the PRL testing phase were identical to the PRL training phase. For the PRL testing, rats were divided into four treatment groups (see the drug treatment section) that were matched based on the performance of individual animals during the baseline criterion sessions.

We recorded several measures of rats’ performance in the PRL task. Firstly, the animals’ decisions were tracked on a trial-by-trial basis in order to monitor their sensitivity to positive and negative feedback. Rewarded outcomes followed by a decision to stay with the lever, which delivered them (win–stay), were counted separately for the correct and incorrect levers and expressed as a ratio of all rewarded outcomes on that lever. This win–stay ratio was used as a measure of sensitivity to either true (correct lever) or misleading (incorrect lever) positive feedback. By the same token, lose–shift ratio was calculated by dividing non-rewarded (i.e., punishing) outcomes after which the animal decided to change the lever, by the total number of punished trials on that lever. This lose–shift ratio represents sensitivity to either true (incorrect lever) or misleading (correct lever) negative feedback.

The ratio of presses on the correct lever to all responses was also analyzed as a measure of the discriminative abilities of the animals. The number of reversals completed during the test was used to assess cognitive flexibility, which relies on the ability to both suppress previously rewarded action and to overcome learned non-reward (Nilsson et al. 2015).

### Drug treatment

Acute doses of ketamine (5, 10, and 20 mg/kg; Biowet, Pulawy, Poland) were administered to rats intraperitoneally 60 min before testing. Furthermore, 24 h and 48 h after the KET administration, the animals were tested again to evaluate a potential long-term effect.

KET (100 mg/ml) was freshly diluted to 5, 10, and 20 mg/ml in saline. Injections were given at a volume of 1 ml/kg. The animals in the control group received the same volume of physiological saline (SAL; 0.9% NaCl).

### Statistical analysis

Raw data recorded by the Med PC software was parsed by means of a custom written R program and analyzed using SPSS (version 21.0, SPSS Inc., Chicago, IL, USA). Differences between experimental groups during 3 baseline sessions were compared using either one-way ANOVAs or the Kruskal–Wallis test, when the data were not normally distributed (as assessed by Shapiro–Wilk normality test). The effects of KET treatment on each measure of interest were compared using two-way repeated measure ANOVAs with a within-subjects factor of test (3 levels: 1, 24, and 48 h after) and a between-subjects factor of treatment (4 levels: saline and 5, 10, and 20 mg/kg). Post hoc pairwise comparisons were performed using Sidak’s correction for multiple comparisons. For all analyses, the significance level was set to *α* = 0.05. Homogeneity of variance was confirmed using Levene’s test and the sphericity was also verified using Mauchly’s test. The data are presented as the mean ± SEM.

## Results

In total, we trained 32 rats, of which 30 fulfilled the criteria to proceed to the testing phase. After establishing the baseline, rats were divided into four groups, which did not differ in their performance levels in any of the measures of interest (lose–shift after misleading negative feedback: *H* = 4185, NS; lose–shift after true negative feedback: *F* (_3, 25_) = 1703, NS; win–stay after misleading positive feedback: *F* (_3, 25_) = 0.3846, NS; win–stay after true positive feedback: *F* (_3, 25_) = 0.2485, NS; Reversals completed: *F* (_3, 25_) = 0.4041, NS; correct lever press ratio: *F* (_3, 25_) = 0.9209, NS). This resulted in the following group-sizes: *n* = 7 (saline control), *n* = 8 (5 mg/kg KET), *n* = 7 (10 mg/kg KET), and *n* = 8 (20 mg/kg KET). One animal from the highest dose group was discarded from analysis, due to a high level of omissions in the 1 h post-injection test; therefore, the final group size for 20 mg/kg KET was *n* = 7.

**Fig. 2:**
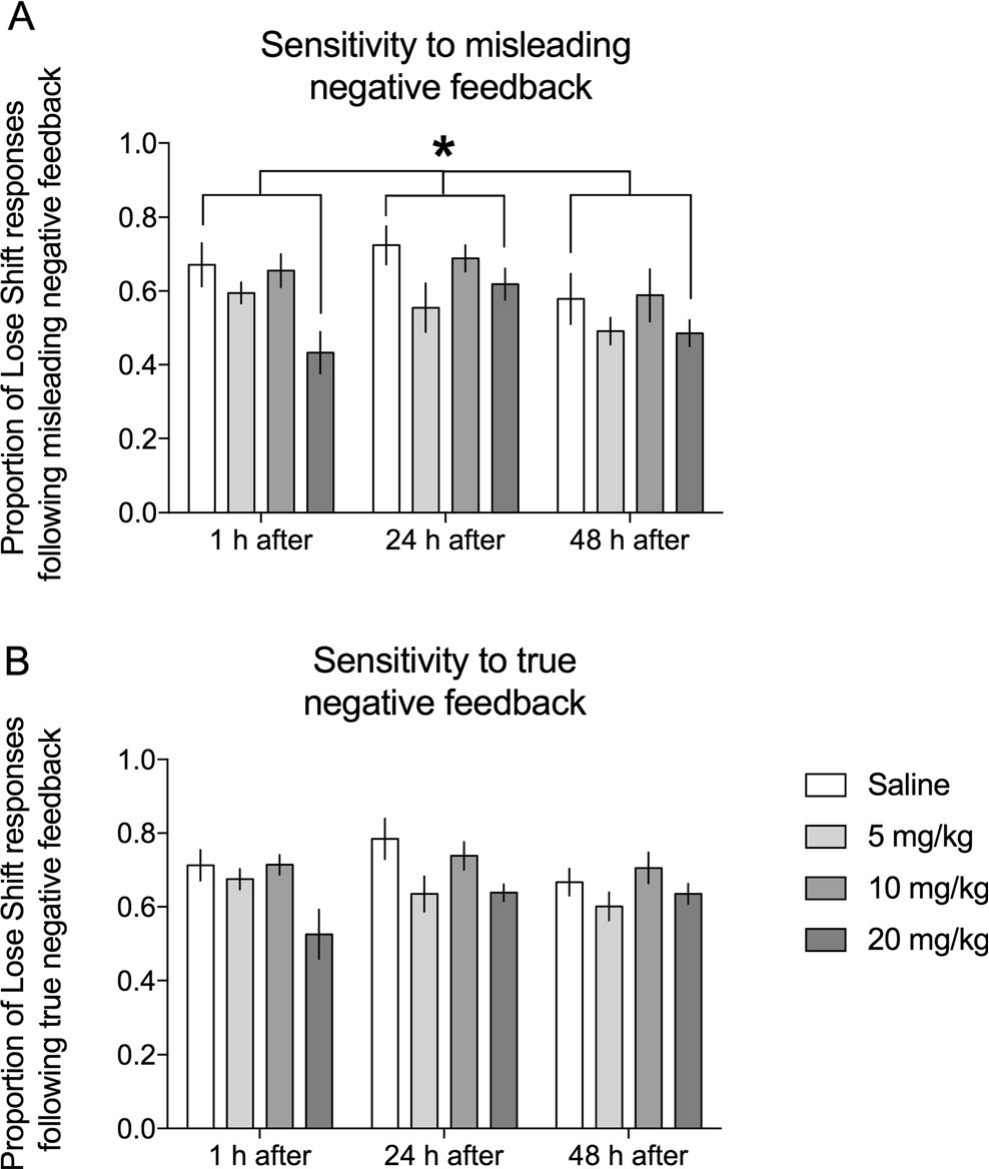
Effects of KET on negative feedback sensitivity. Effects of 3 doses of KET (5, 10, and 20 mg/kg i.p.) on the proportion of lose–shift responses following **a** misleading and **b** true negative feedback in rats tested 1, 24, and 48 h after drug administration. *Asterisk* indicates significant main effect of treatment (*F*_(3,25)_ = 4877, *p* = 0.008) and significant (*p* = 0.025) differences between groups treated with 20 mg/kg KET and physiological saline that were observed regardless of the time of testing (non-significant treatment × test interaction (*F*_(6,50)_ = 1065, NS)). Data are presented as mean ± SEM

Rats injected with the highest (20 mg/kg) dose of KET displayed significantly (*p* = 0.025) lower proportion of lose–shift behaviors following misleading negative feedback compared to the control group (statistically significant main effect of treatment (*F*_(3,25)_ = 4877, *p* = 0.008, Fig. 2a), regardless of the time (1, 24, or 48) after injection (non-significant treatment × test interaction (*F*_(6,50)_ = 1065, NS).

Similar effects were observed following true negative feedback. Rats injected with the highest (20 mg/kg) dose of KET displayed a strong trend (*p* = 0.061) towards a lower proportion of lose–shift behaviors following true negative feedback compared to the control group (statistically significant main effect of treatment (*F*_(3,25)_ = 3898, *p* = 0.021, Fig. 2b), regardless of the time (1, 24, or 48 h) after injection (non-significant treatment × test interaction (*F*_(6,50)_ = 1955, NS).

We did not observe statistically significant effects of lower doses of KET on lose–shift behaviors neither following misleading nor true negative feedback (Figs. 2a, b, respectively).

**Fig. 3:**
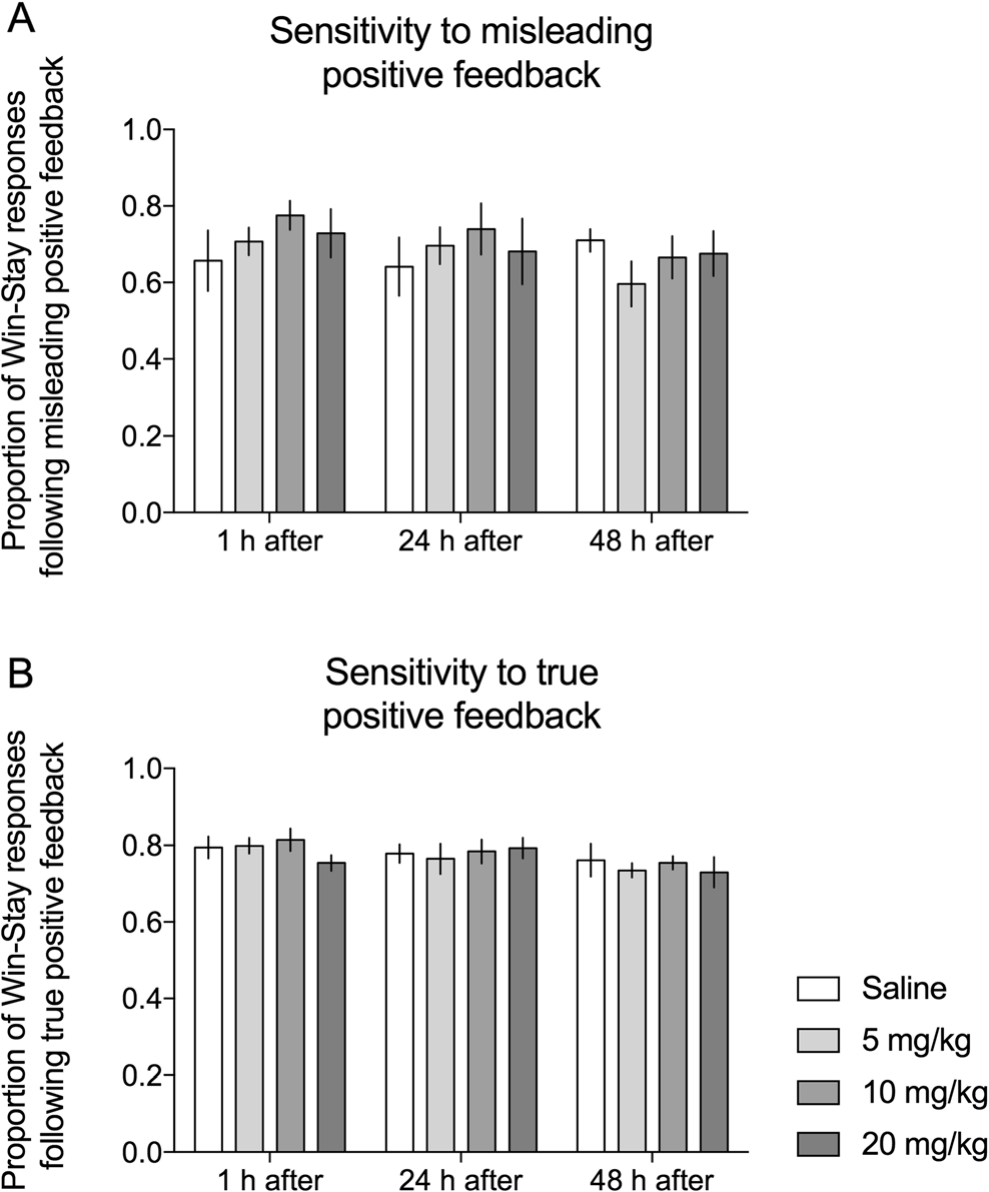
Effects of KET on positive feedback sensitivity. Effects of 3 doses of KET (5, 10, and 20 mg/kg i.p.) on the proportion of win–stay responses following **a** misleading and **b** true positive feedback in rats tested 1, 24, and 48 h after drug administration. Data are presented as mean ± SEM

None of the three tested doses of KET affected positive feedback sensitivity of experimental animals (Figs. 3a, b). Analysis of win–stay behaviors revealed no statistically significant main effects of treatment neither following misleading (*F*_(3,25)_ = 0.249, NS, Fig. 3a) nor true positive feedback (*F*_(3,25)_ = 0.626, NS, Fig. 3b).

**Fig. 4:**
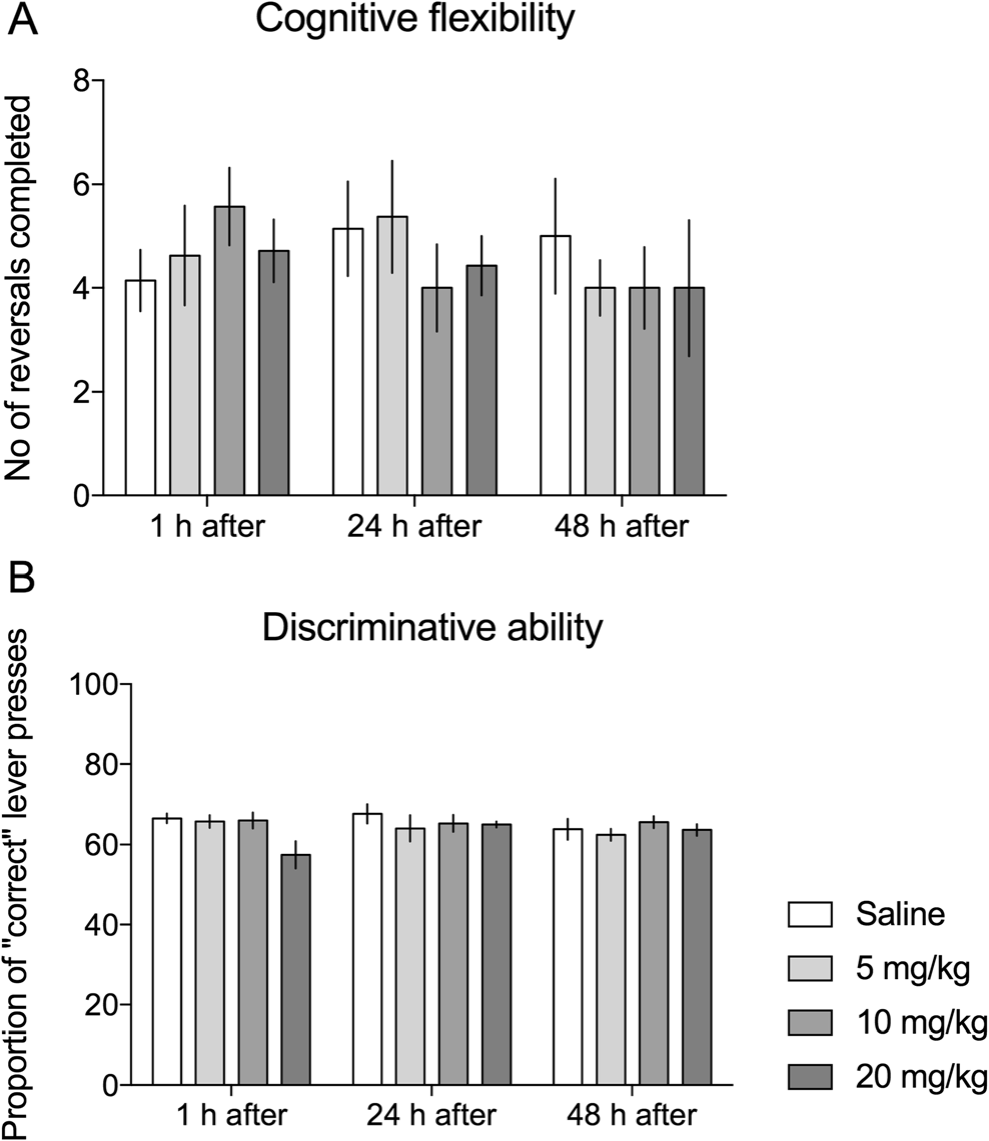
Effects of KET on cognitive flexibility and the discriminative abilities. Effects of 3 doses of KET (5, 10, and 20 mg/kg i.p.) on **a** the number of reversals per testing session, which is a measure of cognitive flexibility, and **b** proportion of correct lever presses, which was used to assess the ability of the experimental animals to discriminate between correct and incorrect levers. Data are presented as mean ± SEM

None of the three tested doses of KET affected cognitive flexibility of experimental animals. Repeated measures two-way ANOVA revealed no statistically significant differences between experimental groups in the numbers of reversals completed (non-significant main effect of treatment: *F*_(3,25)_ = 0.088, NS, Fig. 4a) neither 1 nor 24 or 48 h after KET injection (non-significant treatment × test interaction: *F*_(6,50)_ = 0.6676, NS, Fig. 4a).

KET also did not affect the proportion of correct lever presses (non-significant main effect of treatment: *F*_(3,25)_ = 1537, NS, Fig. 4b) regardless of the time (1, 24, or 48 h) after injection (non-significant treatment × test interaction (*F*_(6,50)_ = 1667, NS, Fig. 4b).

Additionally, KET had no statistically significant effects on motivation of the experimental animals to perform the test, as measured by the mean latency to respond to either correct or incorrect lever (non-significant main effect of treatment for the correct lever: *F*_(3,25)_ = 1710, NS; non-significant main effect of treatment for the incorrect lever: *F*_(3,25)_ = 0.6770, NS) in any of the tested time points (1, 24, or 48 h) (non-significant treatment × test interaction for the correct lever: *F*_(6,50)_ = 1219, NS; non-significant treatment × test interaction for the incorrect lever: *F*_(6,50)_ = 1520, NS).

Moreover, KET did not have a statistically significant sedative effect on tested rats, as there were no differences between the experimental groups in the number of omitted trials (non-significant main effect of treatment: *F*_(3,25)_ = 1668, NS) neither 1 nor 24 or 48 h after drug administration (non-significant treatment × test interaction: (*F*_(6,50)_ = 0.8604, NS).

Lastly, a close inspection of the feedback sensitivity data of control animals during the experimental sessions showed that the pattern of both lose–shift and win–stay responses following true and misleading feedback was similar to the one observed in other rat studies, that utilized the instrumental PRL paradigm (e.g. Dalton et al. 2016). Specifically, the proportion of lose–shift responses following true feedback was always higher than after misleading feedback (lose–shift after true negative feedback: 0.71 ± 0.04, 0.78 ± 0.05, 0.67 ± 0.04 vs lose–shift after misleading negative feedback: 0.67 ± 0.06, 0.72 ± 0.05, 0.58 ± 0.07 1, 24, and 48 h, respectively). Similarly, the proportion of win–stay responses following true feedback was higher than after misleading positive feedback (win–stay after true positive feedback: 0.79 ± 0.03, 0.78 ± 0.02, 0.76 ± 0.04 vs win–stay after misleading positive feedback: 0.66 ± 0.08, 0.64 ± 0.08, 0.71 ± 0.03 1, 24, and 48 h, respectively).

## Discussion

The aim of the present study was to investigate the effects of a non-monoaminergic antidepressant ketamine on feedback sensitivity in rats. We have demonstrated, for the first time, that acute KET treatment significantly diminishes negative feedback sensitivity (NFS) in rats—a cognitive-affective function altered both in depressed individuals (Murphy et al. 2003; Taylor Tavares et al. 2008) and their close relatives (Luking et al. 2016). Similar results were previously obtained with the acute use of an SSRI antidepressant (escitalopram) both in healthy rats (Bari et al. 2010) and mice (Ineichen et al. 2012). The present study further supports the predictive validity of the rodent PRL task, by showing that the test is sensitive to pharmacological manipulation of NFS regardless of the neurotransmitter system targetted by the drug. In the next paragraphs, we discuss the possible neural mechanisms involved in the observed behavioral changes induced by KET.

The reduction of sensitivity to non-reward observed in our study suggests that KET triggered changes in a neural mechanism of non-reward detection. A recent non-reward attractor theory of depression proposed by Rolls (2016) posits that neurons of the lateral orbitofrontal cortex (lOFC) engage in sustained activation after detecting non-reward. The activity of these neurons is either more easily triggered or much stronger in depressed individuals and thus directly leads to overestimating insignificant setbacks (Rolls 2016). Indeed, the work of Quirk et al. (2009) suggests that the neurons in question are the GABA-containing interneurons of lOFC, which are selectively blocked by KET. This observation is in line with one of the possible mechanisms of KET’s rapid antidepressant properties, which is the disinhibition of pyramidal neurons in the PFC (Abdallah et al. 2016). Since lOFC has recently been validated as a region responsible for feedback sensitivity in rats (Dalton et al. 2016), it is plausible that KET-induced reduction of NFS observed in the present experiment was mediated by a glutamate surge in the non-reward-detection network, which would be sufficient to destabilize its function (Rolls 2016).

Roll’s theory of neural basis of depression further states that the symptoms of anhedonia could be explained by the inhibitory action of lOFC network on reward-detection neurons of the medial OFC (mOFC) (Rolls 2016). Therefore, one would expect an effect of KET on positive feedback sensitivity in the present study. Although the mOFC is responsible for feedback sensitivity in rats (Dalton et al. 2016), we did not observe any changes in animals’ reactions to either true or misleading positive feedback after KET. One reason for this might be that rats are highly motivated to obtain reward, due to its high palatability. Animals decide to stay with the recently rewarded option at above chance levels (65–80% of the time), regardless of the current overall reinforcing value of the lever (correct vs incorrect). Because rats exhibit a natural propensity to explore alternative food resources and will never show close to 100% win–stay ratios (which humans do), there might be a “ceiling effect” of reward in the task. However, KET could still be able to reverse stress-induced anhedonia/decrease in positive feedback sensitivity in rats—a possibility worth pursuing in the future studies.

Furthermore, Belujon and Grace (2014) postulated that the antidepressant effects of KET could be caused by the dopaminergic system. The data obtained in the abovementioned study shows that in healthy rats, KET causes both increased firing rates and bursting activity of the VTA neurons, as well as activates D1 receptors in the nucleus accumbens shell. Nucleus accumbens shell selectively mediates positive feedback sensitivity (Dalton et al. 2014), which was not affected by KET in our experiment, possibly due to the abovementioned limitations. According to the dopamine prediction error hypothesis, the absence of an expected reward results in a phasic depression of firing of dopamine-releasing neurons of the VTA (Glimcher 2011; Schultz et al. 1997). Thus, KET could have attenuated the salience of unexpected negative feedback by increasing the firing rates of the VTA (Belujon and Grace 2014) and—subsequently—occluding the phasic “dips”, which signal non-reward.

This additional mechanism could converge with the primary glutamate-driven action of KET during the initial testing session, while the compound was still “on board.” The long-lasting antidepressant effects of KET (although controversial, see Popik et al. (2008)) are thought to arise via homeostatic plasticity changes in the PFC, mediated by activation of synaptogenic intra- and extracellular signaling, including mTORC1 and BDNF pathways (Jernigan et al. 2011; Yang et al. 2013). The release of BDNF is essential for antidepressant effects of KET and remodeling of the PFC networks is compromised in rodent models of depression (Bjorkholm and Monteggia 2016). Therefore, involvement of BDNF in the long-term KET-induced NFS reduction could be tested in the future, for instance by blocking BDNF function in the lOFC. Moreover, additional tests of the possible long-lasting effects of acute monoaminergic antidepressant application on NFS in rodents should be performed, since we do not know if KET shares its profile of action with other antidepressants in this regard. Such experiments would definitely shed some more light on the predictive and face validities of the PRL task.

Recent reports have demonstrated that apart from affecting glutamatergic neurotransmission via NMDARs, KET also potentiates serotonin release in the PFC by amino-3-hydroxy-5-methyl-4-isoxazolepropionic acid receptor (AMPAR) stimulation in the raphe nucleus (Nishitani et al. 2014). In their seminal study, Bari et al. (2010) showed that acute treatment with a high dose of a potent SSRI-citalopram 30 min before a PRL session decreases NFS, while a low dose of the drug has an opposing effect (Bari et al. 2010). Thus, one could hypothesize that serotonergic neurotransmission participated in the results reported herein, especially due to the fact that a high dose of KET administered 60 min before the test had a similar effect to that of a high dose of citalopram (i.e., a decrease in proportion of lose–shift responses following misleading feedback to about 0.40 after acute treatment with both drugs). However, based on the data of Nishitani et al. (2014), the involvement of the abovementioned 5-HT mechanism in KET’s action in the present study could be questioned by the fact that the 5-HT upregulation in the PFC covers the span of 50 min from KET administration (Nishitani et al. 2014), and testing in our study took place after this time had elapsed. Future studies are necessary to elucidate this issue.

The outstanding question is why changes in neurotransmission of brain networks involved in non-reward detection would affect only sensitivity to misleading negative feedback. Our data suggests that the active dose of KET did actually blunt non-reward detection in general. The fact that the drop in true NFS was not low enough to reach significance shows that rats were still able to *learn* from prevalent negative feedback. Furthermore, neither the discriminative abilities of the animals nor the ability to change behavior after reversals was affected by KET. The latter result replicates previous findings (Gastambide et al. 2013; Kos et al. 2011; Nikiforuk et al. 2010), which showed no effect of KET on reversal learning. It is interesting to note that the same treatment in the abovementioned studies had a debilitating effect on set-shifting—another function contributing to cognitive flexibility.

The results of the present study support the predictive validity of the rat PRL task. We have shown that a compound capable of ameliorating symptoms of depression selectively downregulates sensitivity to negative outcomes in rodents. This is a promising result since pathologically elevated negative feedback sensitivity is a hallmark of depression. Moreover, the results of the present experiment are comparable with the only known PRL antidepressant study conducted on rats, with the use of an SSRI drug—citalopram (Bari et al., 2010). Therefore, changes in a basic neurocognitive function of feedback sensitivity could participate in KET’s psychological antidepressant effects; however, its rapid action in humans is possibly driven by the impact of the drug on some other mechanism.

In conclusion, the pleiotropic effects of KET administration modulate complex neuropsychological processes, which can be measured in the PRL task. The key difference between the PRL and other cognitive tasks is that PRL includes an affective and probabilistic decision-making component. This implies that the operant PRL paradigm can grasp the interplay of affect and cognition, which is dysfunctional in psychiatric disorders.

The promise of tapping into subtle cognitive processes and their pharmacological manipulation in animals can only be met by utilizing advanced behavioral paradigms such as the PRL task. Future studies should assess the translational construct validity of the task by testing various rodent depression models, which will hopefully lead to the development of a new addition to a battery of tests aimed at antidepressant screening.
